# Subclinical thyroid dysfunction is associated with adverse prognosis in heart failure patients with reduced ejection fraction

**DOI:** 10.1186/s12872-019-1055-x

**Published:** 2019-04-04

**Authors:** Guodong Yang, Ya Wang, Aiqun Ma, Tingzhong Wang

**Affiliations:** 1 Department of Cardiovascular Medicine, First Affiliated Hospital of Xi’an Jiaotong University, No. 277 West Yanta Road, Xi’an, Shaanxi 710061 People’s Republic of China; 2 Key Laboratory of Molecular Cardiology, Xi’an Jiaotong University, Xi’an, Shaanxi People’s Republic of China; 3 Key Laboratory of Environment and Genes Related to Diseases, Xi’an Jiaotong University, Xi’an, Shaanxi People’s Republic of China

**Keywords:** Subclinical hypothyroidism, Subclinical hyperthyroidism, Heart failure, Prognosis

## Abstract

**Background:**

Subclinical thyroid dysfunction whose typical patterns include subclinical hypothyroidism and subclinical hyperthyroidism, has been indicated to be associated with an increased risk of heart failure (HF). However, the relationship between subclinical thyroid dysfunction and the clinical outcomes of HF patients is uncertain. This meta-analysis was conducted to assess the association between subclinical thyroid dysfunction and the clinical outcomes of HF patients.

**Methods:**

Pubmed, Embase, Web of Science and Cochrane Central Register of Clinical Trials were searched for eligible studies published up to August 1, 2018 which reported the association between subclinical thyroid dysfunction and the clinical outcomes of HF patients. The pooled hazard ratio (HR) with the corresponding 95% confidence interval (CI) was used to assess the association.

**Results:**

Fourteen studies met the eligibility criteria and a total of 21,221 patients with heart failure were included in the meta-analysis. Compared with HF patients with euthyroidism, the pooled HR of subclinical hypothyroidism for all-cause mortality was 1.45 (95% CI 1.26–1.67) in a randomized effects model with mild heterogeneity (I^2^ = 40.1, *P* = 0.073). The pooled HR of subclinical hypothyroidism for cardiac death and/or hospitalization was 1.33 (1.17–1.50) in a randomized effects model with moderate heterogeneity (I^2^ = 69.4, *P* < 0.001). Subclinical hyperthyroid can increase the risk of all-cause mortality without heterogeneity (HR 1.31, 95% CI 1.10–1.55, I^2^ = 25.5%, *P* = 0.225) but have no influence on the risk of cardiac death and/or hospitalization (HR 1.03, 95% CI 0.87–1.23, I^2^ = 0.0%, *P* = 0.958). These significant adverse associations were also retained in subgroup analysis. Sensitivity analysis demonstrated the stability of the results of our meta-analysis.

**Conclusions:**

Both subclinical hypothyroidism and subclinical hyperthyroidism are associated with adverse prognosis in patients with HF. Subclinical thyroid dysfunction may be a useful and promising predictor for the long-term prognosis in HF patients.

## Background

Heart failure (HF) is the end stage of almost all forms of heart diseases and is one of the most common causes of hospitalization and death worldwide [1, 2]. HF patients suffer from a poor prognosis and a high mortality. The mortality of HF patients within 5 years is reported greater than 50% which is higher than in most malignancies [3]. In the past 30 years, though significant progress has been made to treat HF patients, mortality rates are still high [4]. Early risk stratification can accurately identify HF patients with higher risk for adverse clinical outcomes and thus is important for the management of patients with HF.

The poor prognosis of HF is partially due to the influence of comorbidities which include alterations of thyroid function [5–7]. Thyroid hormones have effects on all cells, tissues, and organs in human body and the homeostasis of thyroid hormones is essential to the optimal functioning of the heart [5–7]. Subclinical thyroid dysfunction is common in the adult population. A typical pattern of subclinical thyroid dysfunction include subclinical hypothyroidism and subclinical hyperthyroidism, which is defined biochemically as abnormal serum level of thyroid-stimulating hormone (TSH) with free thyroxine (FT_4_) and free or total triiodothyronine (FT_3_) within their reference range [8, 9]. The prevalence of subclinical hypothyroidism is reported to be 4–20% in the adult population [10–12], and the prevalence of subclinical hyperthyroidism has been reported to be 0.7–9% [11–13]. Increasing studies have shown that both subclinical hypothyroidism and subclinical hyperthyroidism have profoundly impact on cardiac function by modulating heart rate, cardiac contractive and diastolic function, and systemic vascular resistance [5–7]. It has also been acknowledged that both subclinical hypothyroidism and subclinical hyperthyroidism can be a cause of HF and thus the American College of Cardiology/American Heart Association guidelines for the diagnosis and management of heart failure on adults recommend measurement of thyroid function [14]. Though subclinical hypothyroidism and subclinical hyperthyroidism are associated with an increased risk of HF, the relationship between them and the clinical outcomes of HF patients is uncertain. Though several previous studies have investigated the relationship between subclinical hypothyroidism/subclinical hyperthyroidism and the prognosis of HF patients [15–28], the results are inconsistent. Some studies described an increased risk of all-cause mortality or hospitalization for HF patients with subclinical hypothyroidism or subclinical hyperthyroidism but others did not. Considering the small number of HF patients with subclinical hypothyroidism or subclinical hyperthyroidism in most studies, the results may lack statistical power.

In this studies, we performed a meta-analysis to combine the results of all available prospective studies to clarify the relationship between subclinical thyroid dysfunction and the outcomes of HF patients.

## Methods

### Literature search

Two reviewers (GD Yang and Y Wang) searched electronic databases of Pubmed, Embase, Web of Science, and Cochrane Central Register of Clinical Trials independently and all publications up to August 1, 2018 were considered. The search terms used to search potentially relevant studies are as follows: (‘Heart Failure’ OR ‘Cardiac Failure’ OR ‘Myocardial Failure’ OR ‘Heart Decompensation’) AND (‘Hypothyroidism’ OR ‘Hypothyroidisms’ OR ‘Thyroid-Stimulating Hormone Deficiency’ OR ‘TSH Deficiency’ OR ‘TSH Deficiencies’ OR ‘Hyperthyroidism’ OR ‘Hyperthyroid’ OR ‘Hyperthyroids’). In addition, a manual search was conducted by searching relevant bibliography including the references of the reviews on this topic and previously published meta-analysis. The search strategy was without language restriction.

### Inclusion and exclusion criteria

The inclusion criteria are as follows: 1) prospective clinical studies or cohort studies; 2) involved adults (≥18 years old); 3) clear HF with reduced ejection fraction definition which is in accordance with current HF guideline; 4) investigating the relationship between subclinical hypothyroidism/subclinical hyperthyroidism and the outcomes of HF patients; 5) the outcomes of HF patients include all-cause mortality or cardiac death or hospitalization; 6) the hazard ratio (HR) with 95% confidence intervals (95% CI) for subclinical hypothyroidism/subclinical hyperthyroidism and the outcomes of HF patients were reported. Review articles, case reports, meeting abstract and editorials were excluded. We also excluded studies that only reported unadjusted HR or only reported adjusted HR without 95% CIs.

### Study selection

Two independent reviewers (GD Yang and Y Wang) screened the studies using the titles or abstracts or full text to identify eligible studies. Relevant studies were assessed for compliance with the inclusion criteria. Discrepancies and uncertainties were resolved by consensus or by requiring a third author (TZ Wang) to assess it through rechecking the source data and consultation.

### Data extraction

Two authors (GD Yang and Y Wang) conducted data extraction independently using the standardized data-extraction form and a third author (TZ Wang) confirmed the data for their accuracy. The data extracted from the studies include author, study population, country, mean duration of follow-up, mean age, gender percentage, clinical outcomes, adjusted cofounders and the multivariate adjusted HR with the corresponding 95% CI.

### Quality assessment

The quality of the studies was evaluated by two authors (GD Yang and Y Wang) according to a modified scoring system reported previously [29]. Quality assessment was performed according to the following criteria: 1) methods of outcome adjudication and ascertainment accounted for confounders and completeness of follow-up ascertainment; 2) study populations considered a convenience or a population-based sample; 3) appropriate inclusion and exclusion criteria; 4) thyroid function measured more than once; 5) methods of outcome adjudication categorized as use of formal adjudication procedures and adjudication without knowledge of thyroid status; 6) adjustments made for age, sex, New York Heart Association (NYHA) classification, left ventricular ejection fraction (LVEF), and medication; 7) any other adjustments (such as for B-type natriuretic protein [BNP] level, thyroid drug use, and concomitant medication for HF). When a criteria was performed, a score of 1 was given. A score of 0 was given if a criteria was unclear and not achieved. The score ranges from 0 to 7 points where 7 reflects the highest quality.

### Statistical analysis

HR with 95% CI were used to present the pooled effect sizes. I^2^ and Cochran Q statistics were used to evaluate heterogeneity among studies. I^2^ > 50% or *P* < 0.1 indicate the existence of heterogeneity and the random effects model was used. Otherwise, for I^2^ < 50% and *P* > 0.1, the fixed effects model was applied. Subgroup analysis was performed to explore the possible origin of the heterogeneity according to the study quality (≤4 and > 4), ethnicity (United States, Europe and Asia), mean age (≤65 and > 65), mean duration (month) of follow-up (≤24 and > 24), sample size (≤1000 and > 1000), and adjustment for amiodarone or thyroid treatment (Yes and No). Sensitive analysis was also performed by sequentially omitting one study to investigate the influence of a single study on the heterogeneity. Finally, publication bias was illustrated using funnel plot. Begg’s test and Egger’s test were applied to detect the significance of publication bias. Stata 15.0 (Stata Corp LP, College Station, TX, USA) was used for statistical analyses.

## Results

### Search results

**Fig. 1:**
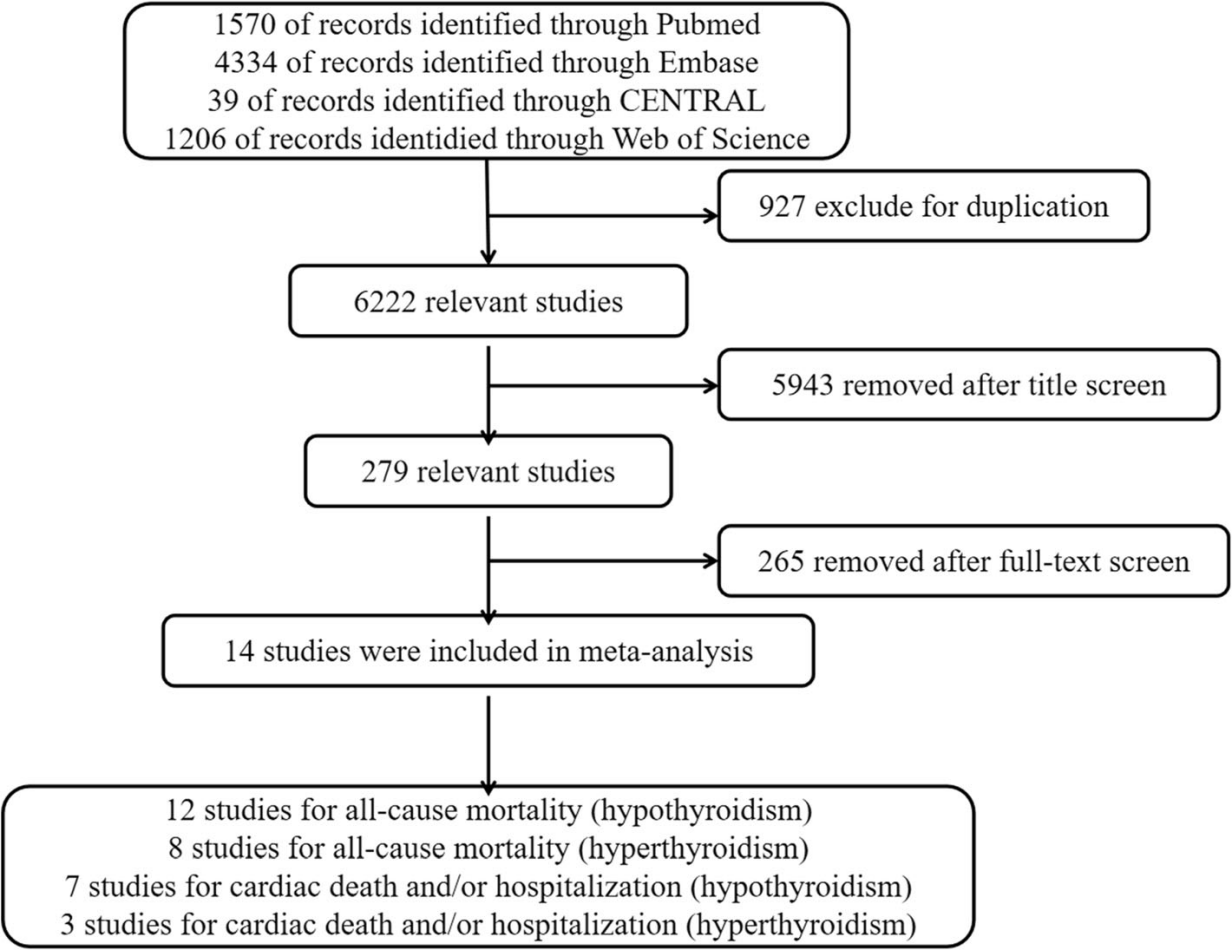
Flow diagram of the selection process

After searching the above electronic databases, a total of 7149 records were obtained. After removing 927 duplicates, 6222 records were screened using title, abstracts and full-texts. Finally, 14 relevant studies [15–28] with a total of 21,221 HF patients were obtained to do meta-analysis. A detailed flow diagram of selecting these relevant studies was presented in Fig. 1.

### Summary of included studies

Table 1 listed the features of the included studies. Twelve studies reported the association between subclinical hypothyroidism and all-cause mortality of HF patients [16, 18–28] and 11 studies reported the association between subclinical hypothyroidism and cardiac death and/or hospitalization of HF patients [15–19, 21–23, 25, 27, 28]. For subclinical hyperthyroidism, 8 studies reported the association with all-cause mortality of HF patients [18, 20–25, 27] and 5 studies reported the association with cardiac death and/or hospitalization [18, 21–23, 27]. The participants in these eligible studies were primarily male and the mean age of the participants ranged from 51 to 72 years old. The follow-up duration of these studies ranged from 12.1 month to 67 month.

**Table 1:** Characteristics of studies included in the meta-analysis

Author (year)	Study population	Country	No. of patients Nor/Hypo/Hyper	Defnition of Hypo/Hyper	Mean follow-up	Mean age (year)	Male %	Outcome	Adjusted variables	Quality score
Iacoviello 2008 [28]	prospective	Italy	304/34/NA	TSH > 5.5mIU/l/ NA	15 mo	64	77	All-cause mortality	Age, sex, BMI, DM, NYHA, HR, hypertension, LVEF, GFR, NT-proBNP, medication	4
Frey 2013 [27]	INH study	Germany	628/34/69	TSH > 4.0 mIU/l/ TSH < 0.3 mIU/l	37 mo	68	71	All-cause mortality	Age	5
Rhee 2013 [26]	NHANES III	United States	410/54/NA	TSH > 4.7 mIU/l	14.3 mo	52.3	42.6	All-cause mortality	Age, sex, race, DM, hypertention, hypercholesterolemia, stroke, MI, BMI, GFR, medication	4
Mitchell 2013 [25]	SCD-HeFT	United States	1930/275/23	TSH > 5.0 mIU/l/ THS < 0.3 mIU/l	45.5 mo	61.3	65	All-cause mortality	Age, sex, DM, renal insufficiency, hypertension, LVEF, time since HF diagnosis, 6-min walk distance, medication	6
Azemi 2013 [24]	Clinical setting	United States	243/102/26	TSH > 5 mIU/l/ TSH < 0.4 mIU/l	27.2 mo	67	77.9	All-cause mortality	Age, sex, TSH, LVEF, DM, primary indication for ICD implantation, medication	5
Deursen 2014 [23]	Observational survey	Italy	2839/290/97	NA/NA	12.1 mo	66	70	All-cause mortality, hospilization	Age, sex, etiology, hypertension, AF, HR, body surface area, systolic blood pressure	4
Chen 2014 [22]	HMO cohort	Israel	4490/916/193	TSH > 4.5 mIU/l/ TSH < 0.45 mIU/l	14.5 mo	75	49	All-cause mortality, cardiac death and hospitalization	Age, sex, DM, ischemic heart disease, hyperlipdaemia, hypertension, AF, BMI, log transformed pulse, log transformed serum urea levels, GFR, hemoglobin, serum sodium, medication	7
Perez 2014 [21]	CORONA	Europe	4338/237/176	TSH > 5.0 mIU/l/ TSH < 0.3 mIU/l	32.8 mo	72	77	All-cause mortality, cardiac death and /or hospitalization	Age, sex, NYHA, LVEF, BMI, BP, HR, MI, smoking, angina pectoris, CABG, PCI, AA, hypertension, BM, AF, ICD, stroke, CPR, medication	6
Li 2014 [20]	Clinical setting	China	816/79/68	TSH > 5.5 mIU/l/ TSH < 0.35 mIU/l	42 mo	52.1	73.7	All-cause mortality	Age, sex, hypertension, AF, drinking and smoking history, QRS duration, LVEF, FT3, T3, T4, NT-Pro-BNP, medication	6
Sharma 2015 [19]	Clinical setting	United States	427/84/NA	TSH > 5.0 mIU/l	36 mo	68	77	All-cause mortality, hospitalization	Sex, creatinine, DM, medication	3
Wang 2015 [18]	Clinical setting	China	353/41/35	TSH > 4.78 mIU/l/ TSH < 0.55 mIU/l	17 mo	51	71	All-cause mortality	Age, sex, BP, NT-Pro BNP, LVEF, smoking, AF, DM, anemia, renal dysfuntion, NYHA, medication	5
Hayashi 2016 [17]	Clinical setting	Japan	188/5/NA	TSH > 4.5 mIU/l	26 mo	70	57	Cardiac death and hospitalization	Age, sex, LVEF, NT-Pro BNP, eGFR	3
Sato 2018 [16]	Clinical setting	Japan	911/132/NA	TSH > 4.0 mIU/l	36.6 mo	68	57.4	All-cause mortality, cardiac death and hospitalization	Age, sex, BMI, BP, HR, NYHA, DM, hypertension, anemia, chronic kidney disease, AF, smoking, LVEF, medication	5
Ro 2018 [15]	Clinical setting	United States	349/25/NA	TSH > 4.7 mIU/l	67 mo	54.5	35	hospitalization	Age, sex, BMI, race, ethnicity, DM, hypertension, hyperlipidemia, CAD, CVD	4

*AF* atrial fibrillation, *BMI* body mass index, *BP* blood pressure, *CABG* coronary artery bypass grafting, *eGFR* chronic heart failure, *HR* heart rate, *ICD* implantable cardioverter, *LVEF* left ventricular ejection fraction, *MI* myocardial infarction, *NYHA* New York Heart Association, *NT-Pro BNP* N-terminal of the prohormone brain natriuretic peptide, *CAD* coronary artery disease, *CVD* cerebrovascular disease, *DM* diabetes mellitus

### Subclinical thyroid dysfunction and HF outcome

**Fig. 2:**
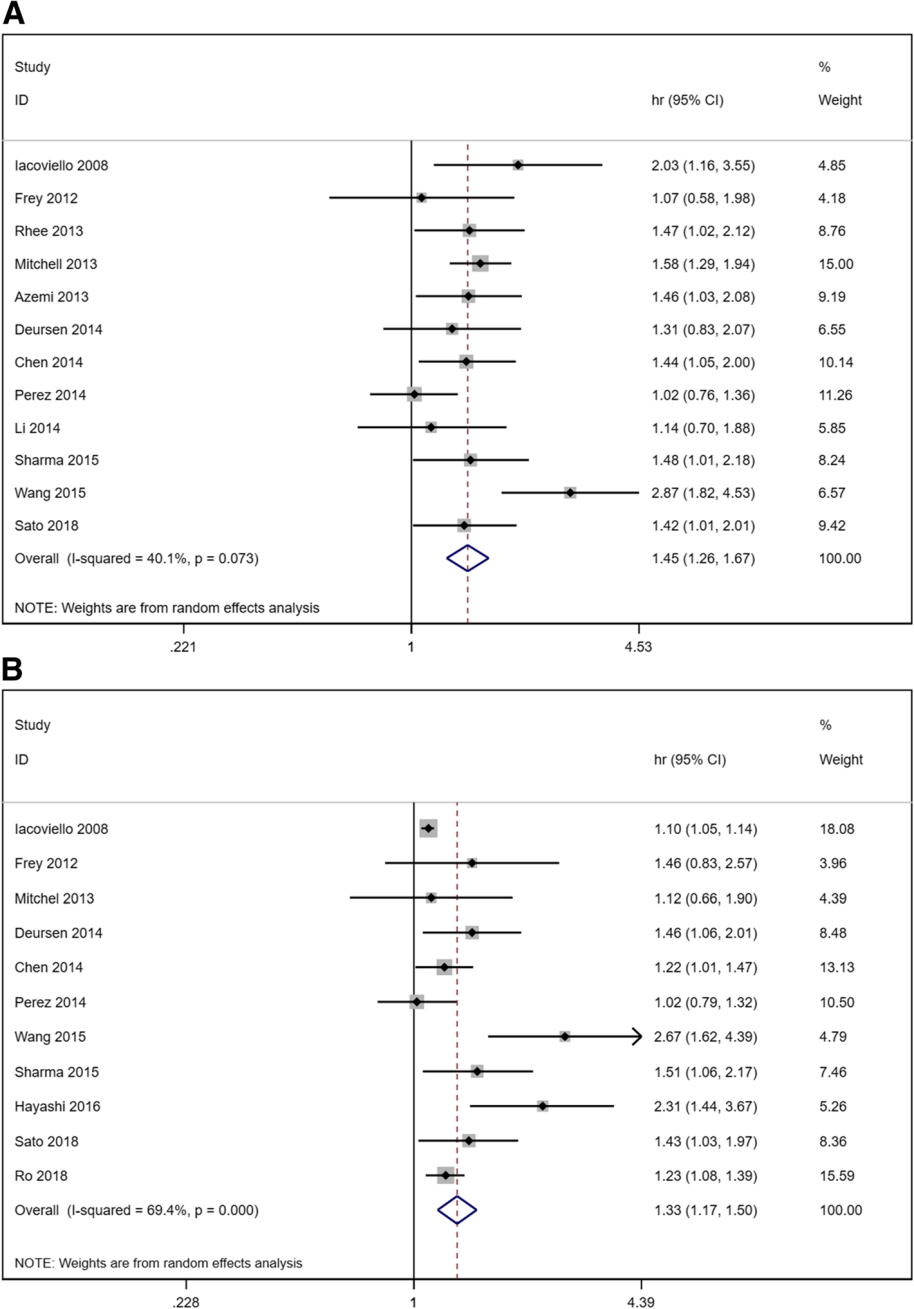
Forest plot of hazard ratio (HR) for hypothyroidism. **a** all-cause mortality. **b** cardiac death and/or hospitalization

**Fig. 3:**
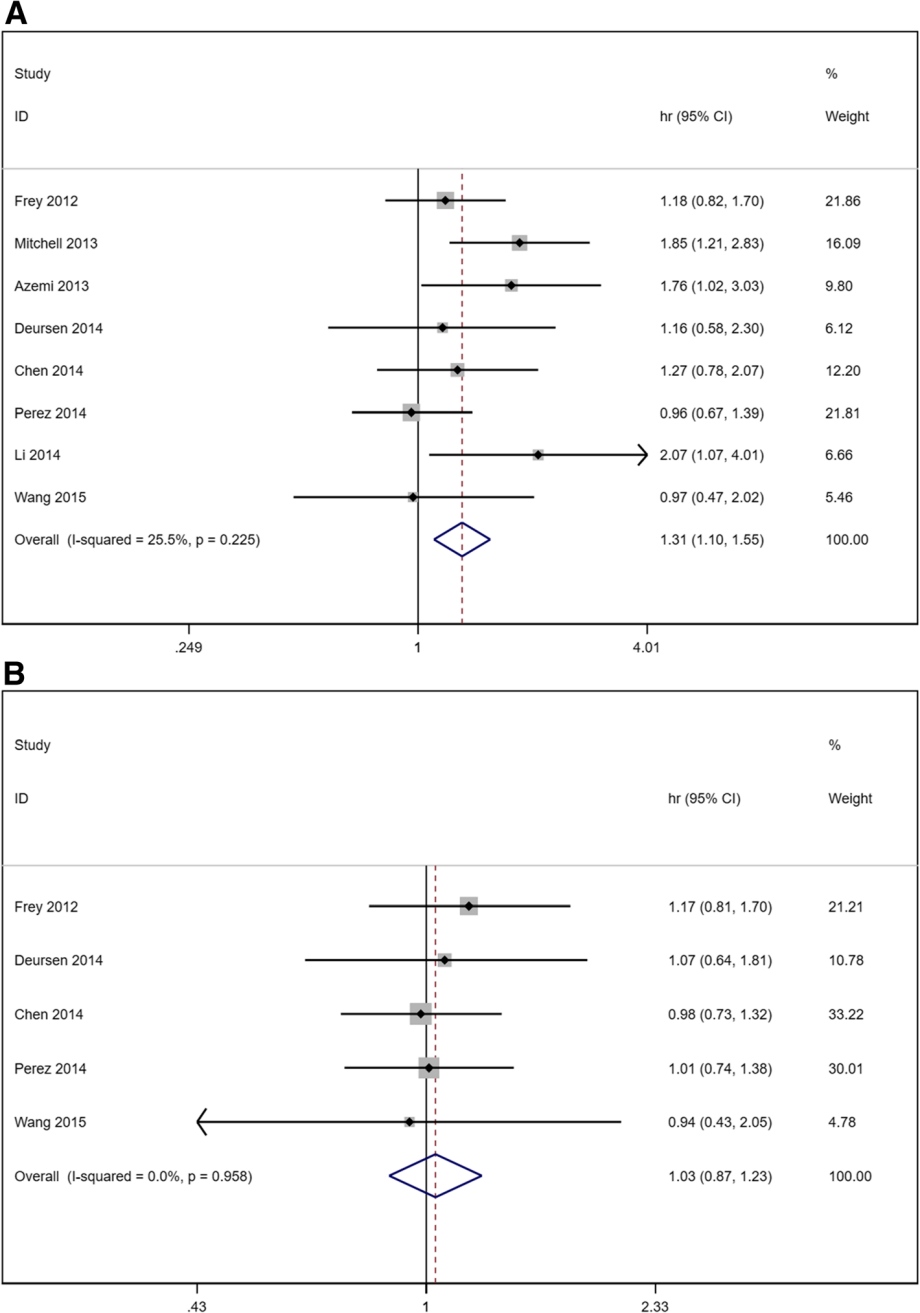
Forest plot of hazard ratio (HR) for hyperthyroidism. **a** all-cause mortality. **b** cardiac death and/or hospitalization

As illustrated in Fig. 2, when compared with patients with euthyroidism, the overall HR of subclinical hypothyroidism for all-cause mortality was 1.45 (1.26–1.67) in a randomized effects model with mild heterogeneity (I^2^ = 40.1, *P* = 0.073). The overall HR of subclinical hypothyroidism for cardiac death and/or hospitalization was 1.33 (1.17–1.50) in a randomized effects model with moderate heterogeneity (I^2^ = 69.4, *P* < 0.001). Figure 3 showed the overall HR of subclinical hyperthyroidism for HF outcome. We can see that subclinical hyperthyroid increases the risk of all-cause mortality without heterogeneity (HR 1.31, 95% CI 1.10–1.55, I^2^ = 25.5%, *P* = 0.225) but have no influence on the risk of cardiac death and/or hospitalization (HR 1.03, 95% CI 0.87–1.23, I^2^ = 0.0%, *P* = 0.958).

### Subgroup analysis and sensitive analysis

A subgroup analysis according to age, ethnicity, mean age, mean duration of follow-up, sample size, score and adjustment of amiodarone or thyroid treatment was performed to investigate the possible origin of the heterogeneity among studies which reported the association between subclinical hypothyroidism and HF. As shown in Table 2, sample size and ethnicity may be the mainly origin of heterogeneity. Besides, our subgroup analysis showed that all-cause mortality had an even stronger relationship with Asian patients (HR 1.67, 95% CI 1.01–2.78) and patients less than 65 years old (HR 1.70, 95% CI 1.31–2.20). In addition, Asian patients also had a stronger association with cardiac death and/or hospitalization (HR 1.76, 95% CI 1.11–2.81).

**Table 2:** Subgroup analysis of the association between hypothyroidism and all-cause mortality or cardiac death and/or hospitalization in heart failure patients

	All-cause mortality					Cardiac death and/or hospitalization				
		Heterogeneity		Meta-analysis			Heterogeneity		Meta-analysis	
Subgroup	Number of studies	I^2^%	*P* value	HR	95% CI	Number of studies	I^2^%	*P* value	HR	95% CI
Age										
≤ 65	5	55.0	0.064	1.70	1.31–2.20	1			1.23	1.08–1.40
> 65	7	0.0	0.603	1.31	1.14–1.50	6	55.3	0.048	1.37	1.14–1.65
Ethnicity										
Europe	6	20.9	0.276	1.31	1.09–1.58	4	32.0	0.220	1.25	1.06–1.47
United States	3	0.0	0.901	1.53	1.31–1.80	1			1.23	1.08–1.40
Asian	3	76.6	0.014	1.67	1.00–2.78	2	63.3	0.099	1.76	1.11–2.81
Follow-up										
≤ 24	5	51.4	0.083	1.70	1.30–2.23	2	0.0	0.343	1.28	1.09–1.50
> 24	7	17.8	0.294	1.35	1.17–1.56	5	62.8	0.030	1.36	1.11–1.66
Sample size										
≤ 1000	7	45.6	0.088	1.57	1.25–1.97	3	72.3	0.027	1.53	1.09–2.15
> 1000	5	33.1	0.201	1.36	1.15–1.61	4	24.7	0.263	1.24	1.07–1.44
Score										
≤4	4	0.0	0.688	1.51	1.22–1.86	4	60.6	0.054	1.48	1.17–1.87
> 4	8	58.2	0.019	1.43	1.18–1.73	3	25.5	0.261	1.32	1.15–1.51
Thyroid drug use										
Yes	5	0.0	0.826	1.48	1.29–1.70	4	56.5	0.075	1.32	1.08–1.60
No	7	64.0	0.011	1.48	1.14–1.94	7	71.8	0.002	1.36	1.12–1.66
Amidarone use										
Yes	6	43.8	0.113	1.31	1.08–1.57	5	46.2	0.115	1.33	1.13–1.56
No	6	19.1	0.289	1.57	1.30–1.90	6	73.7	0.002	1.36	1.09–1.70

**Fig. 4:**
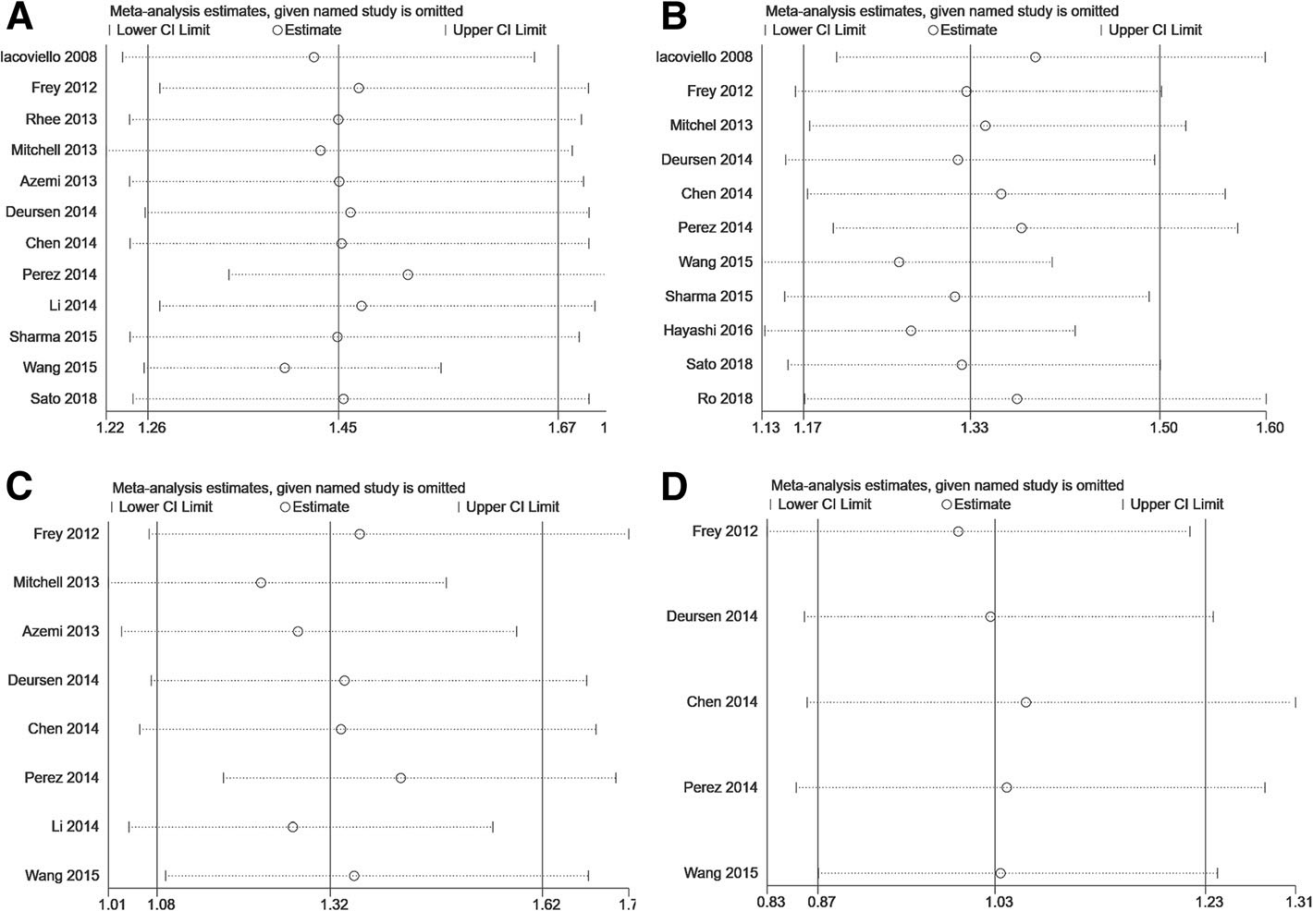
Sensitive analysis. **a** hypothyroidism and all-cause mortality. **b** hypothyroidism and cardiac death and/or hospitalization. **c** hyperthyroidism and all-cause mortality. **d** hyperthyroidism and cardiac death and/or hospitalization

The sensitive analysis was performed by removing one study at a time. Figure 4 illustrated the sensitive analysis. The results didn’t find any study changing the magnitude and direction of the results.

### Publication bias

**Fig. 5:**
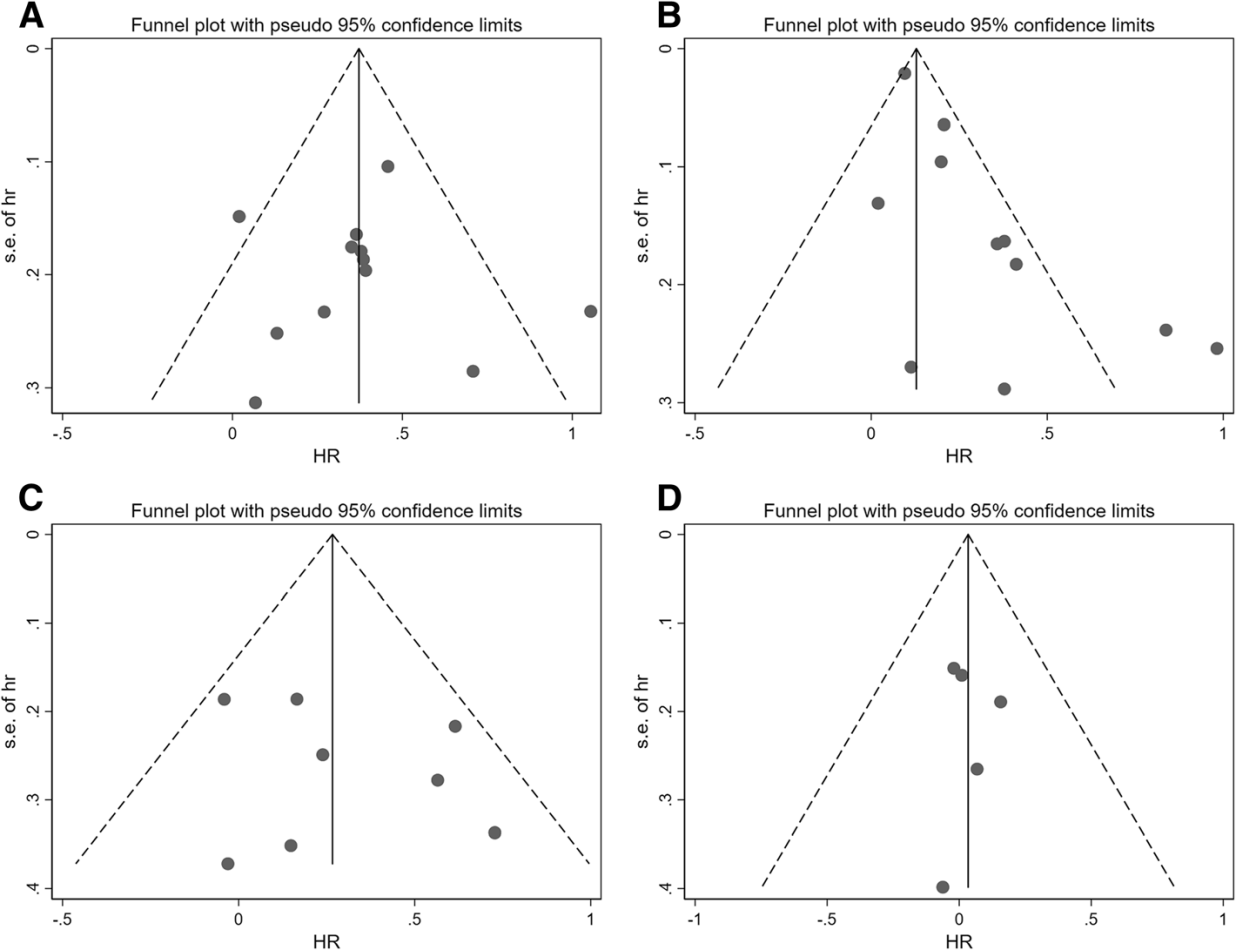
Funnel plot assessing publication bias. **a** hypothyroidism and all-cause mortality. **b** hypothyroidism and cardiac death and/or hospitalization. **c** hyperthyroidism and all-cause mortality. **d** hyperthyroidism and cardiac death and/or hospitalization

We performed funnel plot, Begg’s test and Egger’s test to evaluate the publication bias. The results showed in Fig. 5 and Table 3 indicated there was no publication bias existed among the included studies.

**Table 3:** *P* values of Begg’s and Egger’s test for investigating the publication bias

	Begg’s test	Egger’s test
All cause mortality		
Hypothyroidism	1.00	0.870
Hyperthyroidism	1.00	0.504
Cardiac death and/or hospitalization		
Hypothyroidism	0.119	0.005
Hyperthyroidism	0.806	0.932

## Discussion

The present study demonstrated that both subclinical hypothyroidism and subclinical hyperthyroidism are associated with adverse prognosis in HF patients. Subclinical hypothyroidism can increase the risk of both all-cause mortality and cardiac death and/or hospitality in HF patients. Subclinical hyperthyroidism can also increase the risk of all-cause mortality but appeared to have no distinguishing association with cardiac death and/or hospitality in patients with HF. In addition, these significant adverse associations were also retained in subgroup analysis when adjusting for study quality, ethnicity, mean age, mean duration of follow-up, sample size, amiodarone and thyroid treatment. Besides, sensitivity analysis indicated that no individual study had a remarkable effect on the overall results of the present meta-analysis, demonstrating the results of the current meta-analysis were stable. Considering both subclinical hypothyroidism and subclinical hyperthyroidism are associated with adverse prognosis in HF patients and the test of thyroid function is inexpensive and simple to determine, subclinical thyroid dysfunction may potentially be a useful and promising predictor for the long-term prognosis in HF patients.

In our present meta-analysis, we investigated the association between both subclinical hypothyroidism and subclinical hyperthyroidism and the clinical prognosis in HF patients. A previous meta-analysis published in 2015 [29] has investigated the association between subclinical hypothyroidism and the clinical prognosis in HF patients. Though the previous meta-analysis got the same results as ours, our meta-analysis has some advantages over the previous one. First, this is an update of the previous one. In our meta-analysis, we included four new studies [15–18] which were not contained in the previous meta-analysis and excluded two studies contained in the previous meta-analysis [30, 31] which didn’t report multivariate adjusted HR with 95% CI. Second, our meta-analysis also investigated the association between subclinical hyperthyroidism and the clinical prognosis in HF patients which did not contained in the previous meta-analysis. Third, to our knowledge, this is the first meta-analysis to clarify the relationship between subclinical hyperthyroidism and the outcomes of HF patients. Our results showed that hyperthyroidism can only increase the risk of all-cause mortality but have no influence on cardiac death and/or hospitalization. The number of studies reporting the association between hyperthyroidism and cardiac death and/or hospitalization is relatively small which may lead to a lack of statistical power. Besides, despite the negative effects of hyperthyroidism, there are also potentially positive effects of hyperthyroidism, such as increased contractility [32, 33], reduced peripheral resistance [32, 33], and increased production of natriuretic peptides [34], which may to some extent have compensatory effect.

There are several possible mechanisms accounting for the adverse prognosis of hypothyroidism on HF patients. First, previous studies have reported that hypothyroidism has influence on the structure and function of heart and these alterations can be reversed by thyroid hormone substitutive therapy [35–37]. Second, several studies have reported the link between hypothyroidism and pulmonary hypertension [38–40] which is associated with the mortality with HF patients [41, 42]. Thyroid hormone substitutive therapy can lead to the modification of pulmonary hypertension [38–40]. Third, hypothyroidism can significantly reduce cardiac preload, whereas increasing cardiac afterload results in a consequent reduction in stroke volume and cardiac output [8]. Replacement treatment of thyroid hormone can fully normalized the alterations of hemodynamics [8]. Fourth, hypothyroidism is reported to be associated with anemia which might be one of the causes leading to reduced exercise capacity [43, 44]. Besides the potential mechanisms above, hypothyroidism can also lead to altered lipid metabolism [45], elevated C-reactive protein [46], and increased prevalence of aortic atherosclerosis [47], which can increase the prevalence of myocardial infarction and mortality in HF patients [47, 48].

The potential reasons of why hyperthyroidism is associated with an increased mortality may be as follows. First, hyperthyroidism can cause a high cardiac output state with the increase in heart rate and cardiac preload and the reduced resistance of peripheral vascular [49]. Second, hyperthyroidism is associated with increased heart rate and increased risk of atrial fibrillation [50] which are attributable to the effects of thyroid hormone T_3_ on systolic depolarization and diastolic repolarization with decreased action potential and refractory period duration in atrial and ventricular myocardium [51]. Development of atrial fibrillation may account for increased vascular mortality [51]. In addition, hyperthyroidism is also related to an increased mass of left ventricle [52] which can lead to late diastolic dysfunction [53] and decreased exercise tolerance [54].

There are several strengths of the present study. First, all articles of the eligible cohort studies are published without conference abstract. Moreover, the analysis of included studies is depended on definite inclusion and exclusion criteria. Besides, the present study is an update of the previous meta-analysis investigating the association between subclinical hypothyroidism and the prognosis of HF patients. In the present study, we included four new studies which are not involved in the previous meta-analysis. In addition, to our knowledge, this is the first meta-analysis to investigate the association between subclinical hyperthyroidism and the prognosis of HF patients. The present study also has several limitations. One possible limitation of the present studies is that there is heterogeneity in the included hypothyroidism-related studies. Second, the confounding factors adjusted in different studies are varied. Some well-established variables, such as the history of cardiovascular disease, renal function, natriuretic peptides, and troponins are not adjusted in several included studies. Third, sample size in some included studies is not large enough. Fourth, the number of studies for performing meta-analysis investigating the association of hyperthyroidism and cardiac death and/or hospitalization is relatively small. Because of these general limitations, the results should be interpreted with caution and further studies with larger sample size should be taken to confirm the results.

## Conclusion

The present study demonstrated that both subclinical hypothyroidism and subclinical hyperthyroidism are associated with adverse prognosis in patients with HF. Subclinical thyroid dysfunction may be potentially a useful and promising predictor for the long-term prognosis in HF patients.

## Abbreviations

AF: Atrial fibrillation
BMI: Body mass index
BNP: B-type natriuretic protein
BP: Blood pressure
CABG: Coronary artery bypass grafting
CAD: Coronary artery disease
CI: Confidence interval
CVD: Cerebrovascular disease
DM: Diabetes mellitus
eGFR: Chronic heart failure
FT_3_: Triiodothyronine
FT_4_: Thyroxine
HF: Heart failure
HR: Hazard ratio
HR: Heart rate
ICD: Implantable cardioverter
LVEF: Left ventricular ejection fraction
MI: Myocardial infarction
NT-Pro BNP: N-terminal of the prohormone brain natriuretic peptide
NYHA: New York Heart Association
TSH: Thyroid-stimulating hormone
